# Supplementary material for: Structural Insights and Catalytic Mechanism of 3-Hydroxybutyryl-CoA Dehydrogenase from Faecalibacterium Prausnitzii A2-165
Source: Int J Mol Sci. 2024 Oct 5;25(19):10711. doi: 10.3390/ijms251910711 (PMC11476959; doi:10.3390/ijms251910711)
Supplement: Supplementary file 1 [file ijms-25-10711-s001.zip › ijms-3230414-supplementary.pdf]

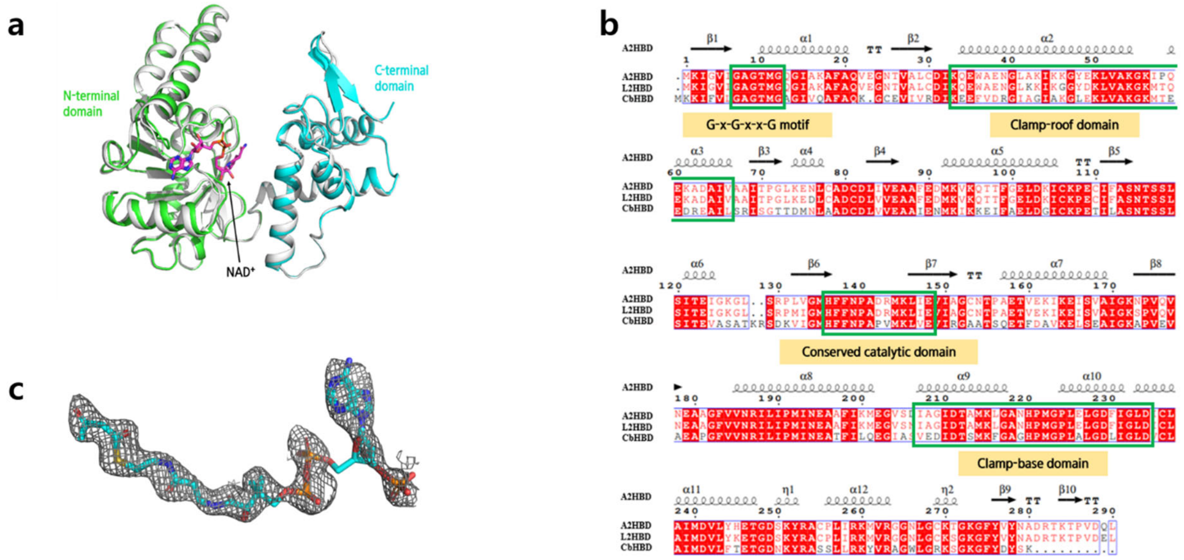

Figure S1. (**a**) The superposition of NAD⁺ complex structures (**b**) Alignment of amino acid sequences of 3-hydroxybutyryl-CoA dehydrogenase of *Faecalibacterium prausnitzii* A2-165, L2-6 and *Clostridium butyricum*. (**c**) The 2fofc electron density map (1.0 σ) of acetoacetyl-CoA by 3rd generation PAL 11C.

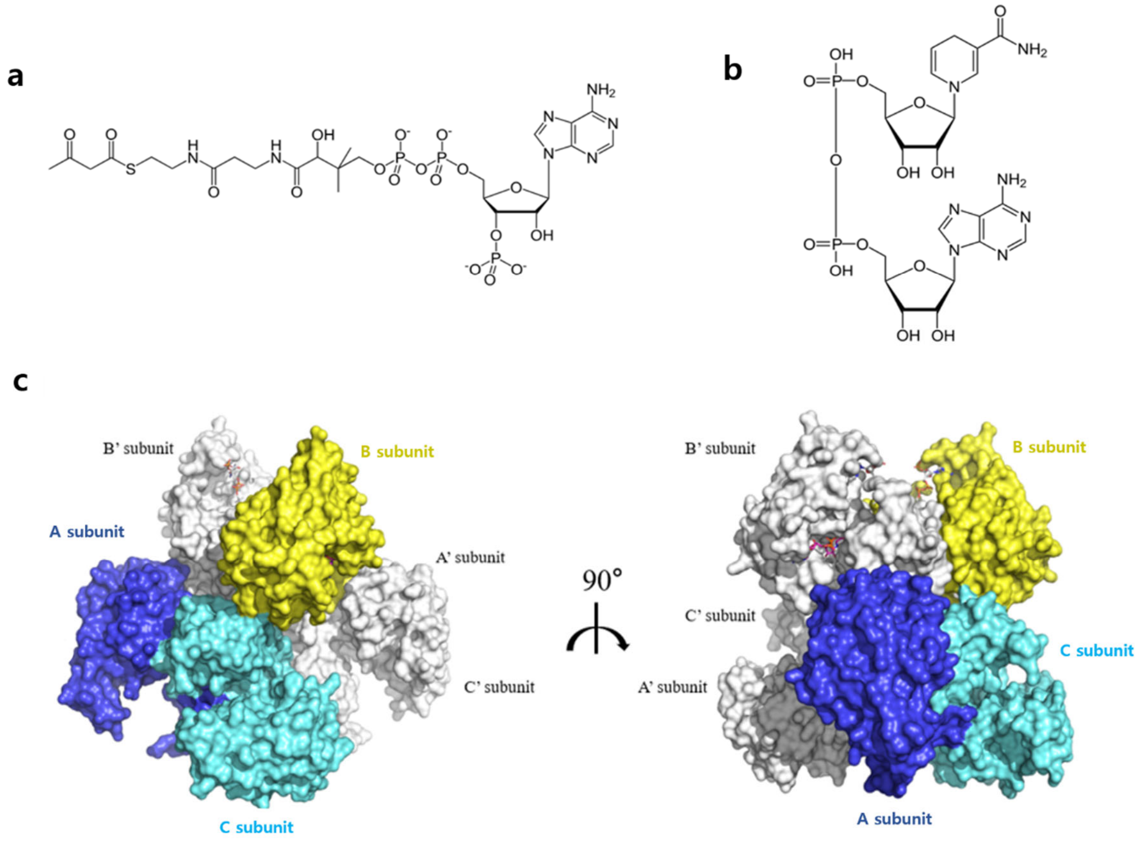

Figure S2. (**a**) Schematic diagram of acetoacetyl-CoA (Substrate) (**b**) NAD⁺ (Cofactor). (**c**) The same structure is shown as the surface model with the same view of Figure 2a.

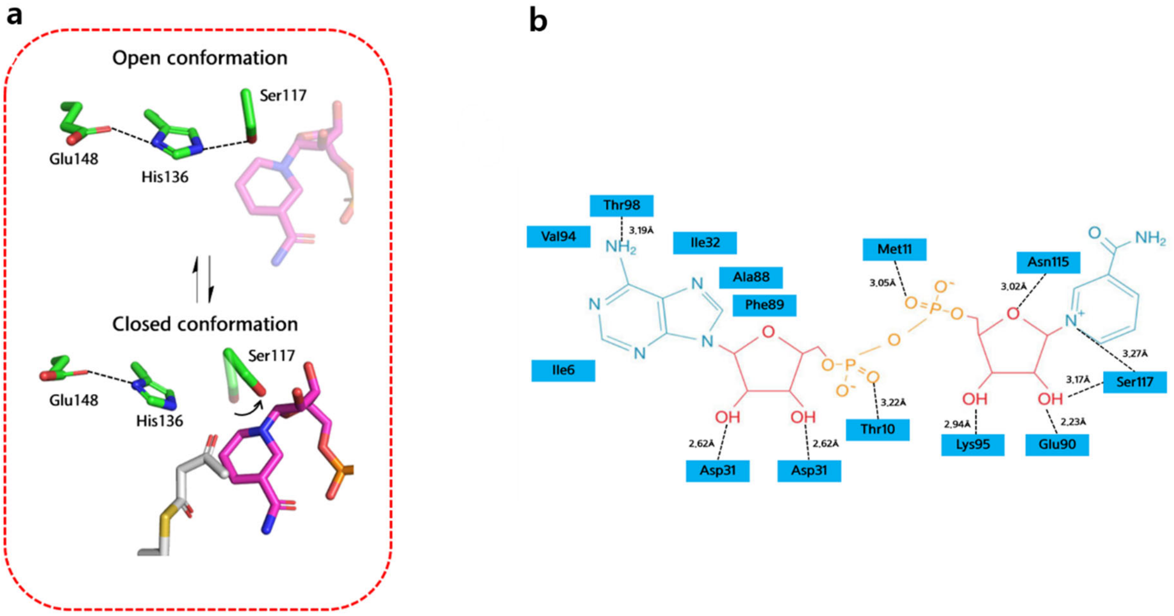

Figure S3. (**a**) Structure-based catalytic mechanism of the acetoacetyl-CoA-catalyzed reaction. (**a**) Hypothesized open-closed conformational change of the catalytic triad (Glu-His-Ser). Hydrogen bonding interactions are shown as black-colored dotted lines. The breakage of the hydrogen bonding interaction between histidine 136 residue and serine 117 residue upon substrate binding is depicted as a curved arrow. (**b**) Schematic interactions between NAD^+ and A2HBD. Hydrogen bonding interactions are indicated as black dashed lines, whereas hydrophobic interactions are not noted in lines.
